# A Semi-Automatic Method for the Quantification of Astrocyte Number and Branching in Bulk Immunohistochemistry Images

**DOI:** 10.3390/ijms24054508

**Published:** 2023-02-24

**Authors:** Sandra I. Marques, Helena Carmo, Félix Carvalho, Susana I. Sá, João Pedro Silva

**Affiliations:** 1 UCIBIO—Applied Molecular Biosciences Unit, Laboratory of Toxicology, Faculty of Pharmacy, University of Porto, 4050-313 Porto, Portugal; 2 i4HB—Institute for Health and Bioeconomy, Faculty of Pharmacy, University of Porto, 4050-313 Porto, Portugal; 3 Unit of Anatomy, Department of Biomedicine, Faculty of Medicine, University of Porto, Alameda Prof. Hernâni Monteiro, 4200-319 Porto, Portugal; 4 CINTESIS@RISE, Faculty of Medicine, University of Porto, Alameda Prof. Hernâni Monteiro, 4200-319 Porto, Portugal

**Keywords:** glial fibrillary acidic protein (GFAP), skeletonize, image analysis, image post-processing

## Abstract

Immunohistochemical staining of cell and molecular targets in brain samples is a powerful tool that can provide valuable information on neurological mechanisms. However, post-processing of photomicrographs acquired after 3,3′-Diaminobenzidine (DAB) staining is particularly challenging due to the complexity associated with the size, samples number, analyzed targets, image quality, and even the subjectivity inherent to the analysis by different users. Conventionally, this analysis relies on the manual quantification of distinct parameters (e.g., the number and size of cells and the number and length of cell branching) in a large set of images. These represent extremely time-consuming and complex tasks, defaulting the processing of high amounts of information. Here we describe an improved semi-automatic method to quantify glial fibrillary acidic protein (GFAP)-labelled astrocytes in immunohistochemistry images of rat brains, at magnifications as low as 20×. This method is a straightforward adaptation of the Young & Morrison method, using ImageJ’s plugin Skeletonize, coupled with intuitive data processing in datasheet-based software. It allows swifter and more efficient post-processing of brain tissue samples, regarding astrocyte size and number quantification, the total area occupied, as well as astrocyte branching and branch length (indicators of astrocyte activation), thus contributing to better understand the possible inflammatory response developed by astrocytes.

## 1. Introduction

Astrocytes are glial cells representing a prominent component of the central nervous system (CNS), which participate in tissue homeostasis throughout the several stages of development, injury, and infection, as well as in the modulation of neuronal and immune responses [1,2,3]. Astrocytes have a star-shaped morphology and express the glial fibrillary acidic protein (GFAP). GFAP is increasingly expressed upon an insult to the tissue [1,4]. This protein thus may be used as a suitable biomarker in immunohistochemistry (IHC) to assess the homeostasis of the tissue, by allowing the quantification of the number of astrocytes, as well as their branching, and branches’ length.

Nevertheless, quantification of IHC photomicrographs is challenging, as

**Figure 1:**
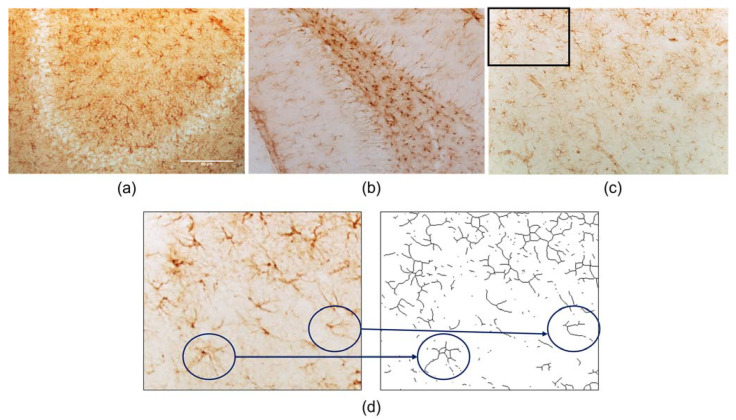
DAB-stained GFAP IHC photomicrographs, acquisition with 20× magnification. (**a**) CA3 zone of hippocampal formation; (**b**) dentate gyrus and hilus zones from the hippocampal formation; (**c**) prefrontal cortex zone with inset drawn; (**d**) detail from the inset of image (**c**), from the prefrontal cortex, and skeletonized detail of the inset, blue highlights feature the conversation of the pointed astrocytes into the skeletonized version. Bar = 50 µm.

**Figure 2:**
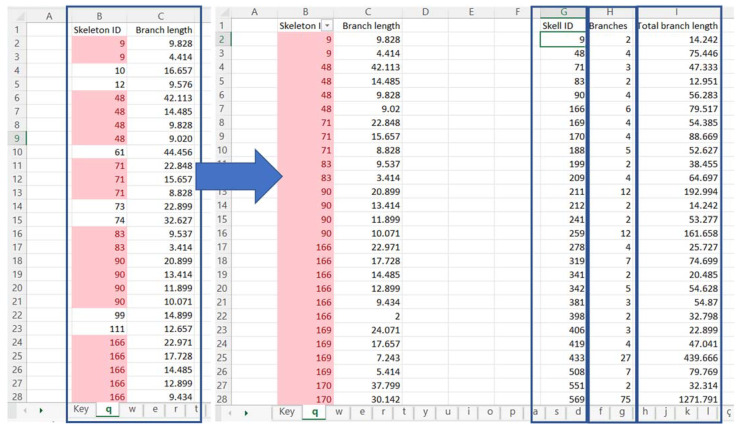
Microsoft Excel table representation of area analysis. “Skeleton ID” (column B) and “Branch length” (column C) columns were retrieved from the “Branch information” result table from ImageJ; followed by Skeleton ID column post single entries filtering, and data organization by each skeleton ID, quantification of the number of branches present in each cell and quantification of the total length of branches for each skeleton.

(1) the distinction between cells and branches is hindered by the large number of cells per area, and the superposition of astrocytes’ branches superposition with other cells’ bodies and branches (Figure 1a–c); (2) 3,3′-Diaminobenzidine (DAB) staining is not stochiometric, since the brown staining is a product of DAB oxidation by horseradish peroxidase, and its amount is correlated to the reaction time (which may, in turn, be influenced by factors like temperature or enzyme amounts); (3) sample groups have to be large enough to attain any statistical significance; and (4) it is highly dependent on photomicrographs’ quality and magnification, as in, the above mentioned, DAB quality reaction, target density, and microscope quality/settings, may influence the image characteristics, which, in turn, directly influence a user’s visual scoring. Classical quantification methods are usually based on visual scoring, rendering them highly subjective, time-consuming, and relying on features such as area occupancy [5,6], while branch quantification is highly dependent on the photomicrograph’s magnification and quality [7]. Altogether, these issues hamper the proper analysis of large amounts of photomicrographs, often taken with a 20× magnification. Previously, Young & Morison developed a method based on the use of the ImageJ software and its plugin Skeletonize (Figure 1d), that may be applied to both IHC and immunofluorescence involving DAB staining [8]. Still, this method was not developed to be applied to low magnification photomicrographs, presenting a high number of cells, nor does it explore the data trimming post-image analysis. Here, we describe a reliable, and inexpensive method, adapted from Young & Morison, to specifically quantify, in a semi-automatic manner, DAB-stained astrocytes in IHC, by applying a threshold algorithm fitter to astrocytes’ quantification [9]. Of note, this improved method requires minimal image post-processing before its analysis, as it does not require any changes to brightness and contrast. Here, we present a detailed explanation of the steps taken to quantify GFAP-stained astrocytes, from creating a quantifiable mask in the photomicrographs, using ImageJ, to the mathematical analysis of ImageJ data and its graphical representation.

## 2. Results

### 2.1. Analysis of Photomicrographs from Each Brain Area

The analysis ran by AnalyzeSkeleton is presented within two data tables per image (“Branch information” and “Results”), which may be saved in a datasheet-based software, such as Microsoft Excel or MATLAB. In our case, only the “Branch information” table was considered of interest for further analysis. From each “Branch information” table, the “Skeleton ID” and “Branch length” columns were exported to a new spreadsheet (Figure 2). Each sheet in the spreadsheet document should represent a single image of the same anatomical area and animal.

A filter was applied to the “Skeleton ID” column [select column B > data tab > filter, define filter for double entries] to select and remove non-duplicated values. This process allowed us to identify and remove all singular entries, as cells with only one branch were not considered to be astrocytes.

Afterward, data was organized into 3 columns:

- A column with the remaining Skeleton IDs, without the duplicates (Figure 2, column G) [copy column B, paste it on column G, select new column Data tab > Delete duplicates].
- A column with the number of branches each Skeleton ID has (Figure 2, column H) [insert the function COUNTIF, in which the first condition comprises the values in column B, and the second one is the cell identification from column G, e.g., =COUNTIF($B$2:$B$5000:G2)].
- And a column for the sum of each Skeleton’s branches length (Figure 2, column I) [insert the function SUMIF, in which the first condition is the values in column B, the second is the cell identification from column G and the third condition is Column C, e.g., =SUMIF($B$2:$B$5000;G2;$C$2:$C$5000)].

**Figure 3:**
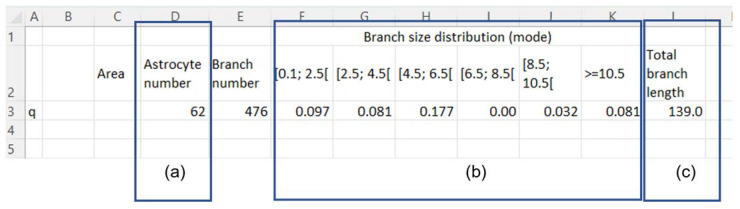
Microsoft Excel table representation for “Key” table—meant to summarize the information of each image. (**a**) Sum of the total number of cells; (**b**) Distribution of the number of branches, according to pre-defined length range; (**c**) sum of the total length of the branches of all cells.

In our analysis, each zone comprised several images, so we combined the information of each image analysis into a sum of the information, representing the zone. For this purpose, a ‘key’ table was designed to retrieve and organize the information from the sheet of each image into a table comprising (Figure 3):

- The sum of the number of cells from each image (Figure 3a);
- The mathematical mode distribution of the branches’ size, normalized to the number of cells (Figure 3b);
- The sum of the total length of the branches (Figure 3c).

### 2.2. Groups’ Analysis Per Area

**Figure 4:**
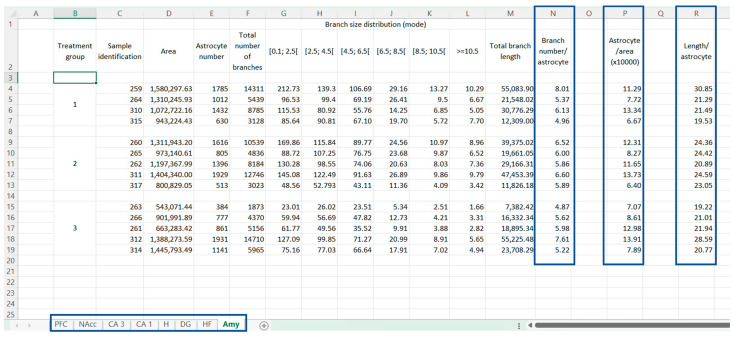
Representation of Group analysis. Table summarizes the information (as a sum) of each sample, organizing it according to sample treatment. (Column N) Normalization of the number of branches per number of cells; (column P) Normalization of the number of cells per area and (column R) normalization of the total branch length per cell.

The comparison of the information per animal was performed in a table (Figure 4), in which we further determined:

- The number of branches per cell (Figure 4, column N), given by the [total branch number (column F) divided by the number of astrocytes (column E)];
- The number of cells per area (Figure 4, column P), calculated as the [number of astrocytes (column E) divided by the area (column D)];
- And the normalization of the total branch length per cell (Figure 4, column R), was obtained by the [normalization of the total branch length (column M) and by the astrocyte number (column E)].

### 2.3. Graphical Representation Data

**Figure 5:**
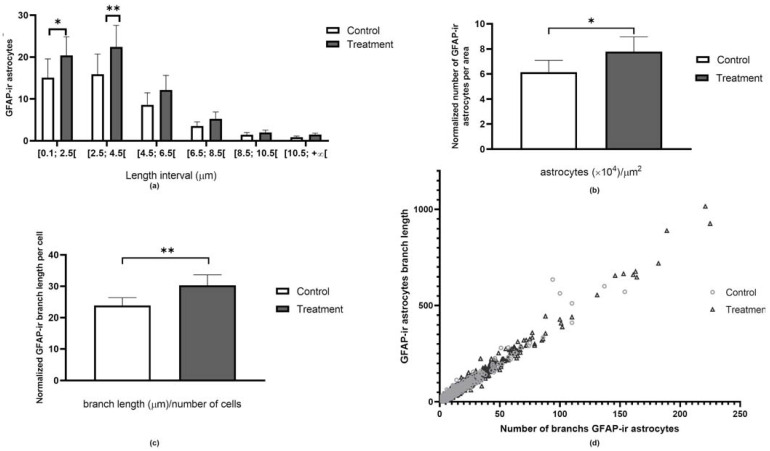
Graphical representation of the data after Excel processing. (**a**) Mode—distribution of the normalized number of branches according to defined size intervals; (**b**) Normalized number of cells per area; (**c**) Normalization of branch length per number of cells and (**d**) Scatter-plot representing each cell according to the number of branches and their respective total size. * *p* < 0.05, ** *p* < 0.01, compared to the control.

Based on the information from the spreadsheet analysis, four main parameters can be represented as:

1. A mathematical model (Figure 5a) representing the most frequent number of the branches’ length, distributed by length intervals, and normalized by the cell number. An increased branch size comparatively to the control group is a potential marker of an inflammatory state, since branching and branch size are known to increase upon astrocyte activation [10];
2. The number of astrocytes (Figure 5b), normalized per area. The fluctuation in the number of cells present in a tissue is a marker for tissue homeostasis changes;
3. The mean branch length per cell (Figure 5c). The general increase in the branch length is another indicator of possible changes to tissue homeostasis;
4. A correlation between the number of branches and their length that can be projected in a scatter-plot (Figure 5d), as a virtual cell size. This graphical representation provides a spatial perception of the increasing astrocyte size/activation, among treatments, visually providing more information about a possible change in the homeostasis of the tissue.

## 3. Discussion

Throughout the last decades, astrocytes have evolved from being considered stationary cells of the central nervous system (CNS), into being paramount supporters of neuronal architecture and function. They have been demonstrated to actively participate in, for example, the blood-brain barrier control, neuronal development, neuro-transmitter turnover, synaptogenesis, and immunological response [1,3,11,12].

With such a broad spectrum of functions, it becomes clear that the assessment of astrocytes’ number and branches is a relevant biomarker to assess the homeostasis of the tissue. In this sense, the development of a quantification method that allows the analysis of a large number of samples of DAB-stained GFAP photomicrographs, in a fast, reliable and reproducible way has gained the utmost importance. Here, we presented a method that relies on free, straightforward, macro-friendly, open-source programs (e.g., ImageJ), and a spreadsheet platform (e.g., Microsoft Excel, MATLAB). Noteworthy, ImageJ is a well-established tool within the scientific community that does not require knowledge of complex programming language, thus being more accessible to a larger population of researchers.

This method gathers important characteristics such as the ability to determine the number and branching of cells, in an accurate manner, without the need to rely on an expensive microscope equipped for stereology or user-biased manual quantification, being reliable even when applied to photomicrographs with a magnification as low as 20×. This results in a tool capable of accurately quantifying samples that arise from commonly used laboratory staining techniques, only requiring a basic light microscope, coupled to a camera, without needing any specific extra hardware or software. The main advantages of the method herein developed, relative to the conventional analysis of photomicrographs are summarized in Table 1.

As with any other method, we are aware that this method presents some limitations. For example, this method is especially adapted to quantify DAB-stained GFAP photomicrographs, in which cell’ size does not change throughout activation. Therefore, it is not ideal to quantify, for example, CD11b-expressing glia cells (i.e., microglia), since these cells change their shape upon activation. Nevertheless, it is possible to adapt it to allow the accurate quantification of such cells. Also, the branch quantification component is not compatible with non-branching-stained targets, such as receptors, or intracellular components.

In sum, this method is a reliable, fast, and reproducible tool that can be used to quantify DAB-stained GFAP samples in bulk. It is a straightforward, free, and macro-friendly adaptation of hardware and software already existing in a functioning laboratory. With it, the quantification of the number and branching of astrocytes in sample tissue becomes a simple task, which should not be underrated since astrocytes are extremely important participants in tissue development and homeostasis. Moreover, this method can be applied to accurately quantify any non-cell shape-changing, branching cells.

## 4. Materials and Methods

### 4.1. Samples

All experiments were performed on drug-naïve male Sprague-Dawley rats (250–300 g, Taconic, Lille Skensved, Denmark) according to guidelines from the Swedish National Board for Laboratory Animals approved by the Animal Ethics Commit-tee of Uppsala, Sweden (ethical approval Dnr 5.8.18-12230-2019). All rats were housed in groups at 20 to 22 °C under a 12-h light/dark cycle with ad libitum access to food and water. Brain tissue samples, isolated from Sprague-Dawley rats, were obtained from Prof. Miroslav Savić’s lab (Belgrade University, Beograd, Serbia).

Briefly, whole brains were collected following the animals’ perfusion with a saline solution, followed by 4% paraformaldehyde. The animals were decapitated, and the brains removed from the skull, washed three times in a phosphate buffer, for 10 min each. The brains were then post-fixed in the same solutions for 30 min, placed in a series of sucrose solutions (with increasing concentrations up to 30%) and then stored at −20 °C in OLMOS solution [6]. For immunohistochemistry, the hemispheres were separated by a mid-sagittal cut and 35 µm-thick coronal sections were sliced from each hemisphere in a vibratome, according to a protocol previously described by Dias-Carvalho et al. (2022) [6].

### 4.2. Immunohistochemisstry

Immunohistochemistry processing was done as previously described [6]. Briefly, samples were recovered from OLMOS solution, washed with 0.01 M phosphate-buffered saline (PBS), treated for endogenous peroxidase inactivation with 10% hydrogen peroxidase (H_2_O_2_) in PBS and blocked with 5% normal serum (Vector Laboratories). In free-floating, primary antibody incubation was performed at 4 °C for 72 h, 1:1000 dilution, in PBS with 0.5% Triton X-100 (polyclonal antibody rabbit anti-GFAP; Z0334, AB_10013382, Agilent Dako, Carpinteria, CA, USA). At room temperature, the secondary antibody, anti-rabbit IgG biotinylated antibody (BA-1100, Vector Laboratories, Burlin-game, CA, USA) was incubated for 1 h, followed by avidin-biotin complex (PK-6100, Vectastain Elite ABC Kit; Vector Laboratories) for 1 h, and 0.05% DAB/ 0.01% H_2_O_2_ in PBS revelation. DAB reaction was stopped with PBS and sections were mounted in gelatin-coated slides, finishing the preparations with histomount mounting media (National Diagnostics, Atlanta, GA, USA).

### 4.3. Image Analysis

Photomicrographs of different brain areas (e.g., hippocampal formation, prefrontal cortex and nucleus accumbens) were acquired using a Zeiss AXIO Imager 2, with a 20× objective, and the Axiovision 40v software, and further converted to JPG format.

Photomicrograph analysis was performed using ImageJ software (FIJI or ImageJ https://imagej.net) and the plugin Skeletonize [AnalyzeSkeleton (2D > 3D) <http://imagej.net > AnalyzeSkeleton> (accessed on 24 February 2022)] [13]. After transforming the photomicrographs of GFAP-stained astrocytes into automatically quantifiable masks to extract the max amount of information possible, this analytical software presents the proper tools to process these images using the subsequently described steps.

To calibrate ImageJ into micrometers, the image scale was set using the straight-line selection tool to draw a line over an existing scale bar in the image, and then selecting [Analyze > Set Scale]. The option [global] was selected to apply the scale settings to the whole set of images. This step ensures that the area and branch length information is accurately measured.

The photomicrographs are loaded into ImageJ, converted to an 8-bit format [Image > Type > 8-bit], applied the FFT bandpass filter [Process > FFT > bandpass filter] and transformed to grey-scale [Image > Lookup tables > greys]. Some of the image processing performed is only possible with 8-bit and grey-scale converted images. The FFT bandpass filter clears small features (i.e., noise) from the image without changing the larger features (e.g., cells and branches). Of note, the ImageJ’s default settings are adequate for DAB-stained GFAP photomicrographs, so no changes in the FFT settings are required.

The Unsharp mask [Process > Filters > Unsharp mask] and Despeckle were then applied [Process > Noise > Despeckle]. Once again, ImageJ settings are appropriate: application of the unsharp mask, and changing the settings, may result in a more fragmented mask of the areas of interest, and Despeckle is related to the removal of salt and pepper noise.

**Figure 6:**
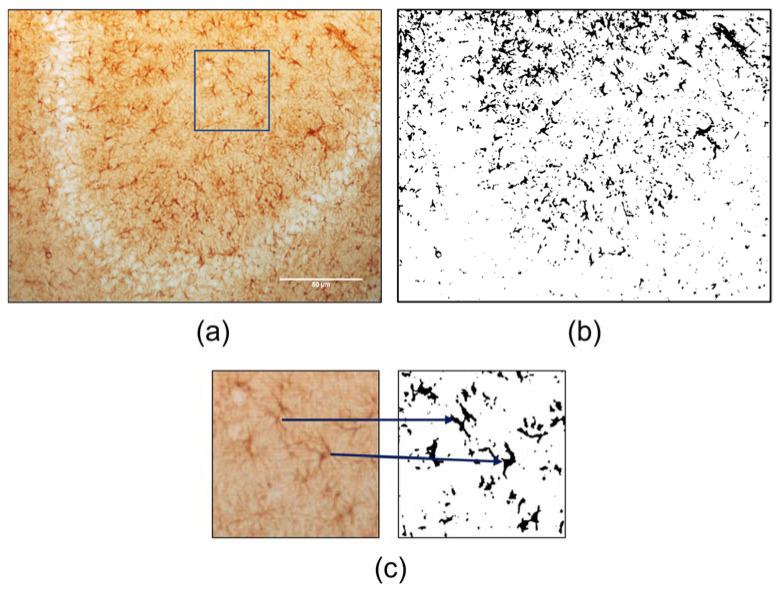
Representative regions of the same photomicrograph through the processing steps. (**a**) Photomicrograph from hippocampal formation CA 3 zone, acquired with 20× magnification; (**b**) Post-processing mask, after application of threshold tool; (**c**) detailed inset from image (**a**) and corresponding detailed mask from image (**b**), blue arrows highlighting the detail between selected GFAP stained cells and threshold recognition. Bar = 50 µm.

After this image pre-processing, the Threshold tool was applied [Image > Adjust > Threshold]. The algorithm was set to MaxEnthropy, the option black background was selected, and the image was further converted to mask (Figure 6a,b). The MaxEnthropy algorithm is the most suitable, among the available algorithms, for astrocyte analysis, as suggested by Siritantikorn et al. (2012) [9]. Defining the black background option false ensures that the background of the image is not considered by the skeletonize plugin.

After conversion of the photomicrographs into the mask, Despeckle [Process > Noise > Despeckle] was reapplied, followed by the Close option [Process > Binary> Close]. The application of the Despeckle option again clears the salt and pepper noise resulting from the mask conversion, whereas the Close option connects dark pixels that are separated by 2 white pixels, uniformizing the open particles.

Then, outliers were removed [Process > Noise > Remove Outliers]. The pixel radius was set to 2 and the threshold to 50. ImageJ calculates the median pixel of the radius and replaces the value of a pixel that falls outside the defined threshold, that deviates from the mean.

**Figure 7:**
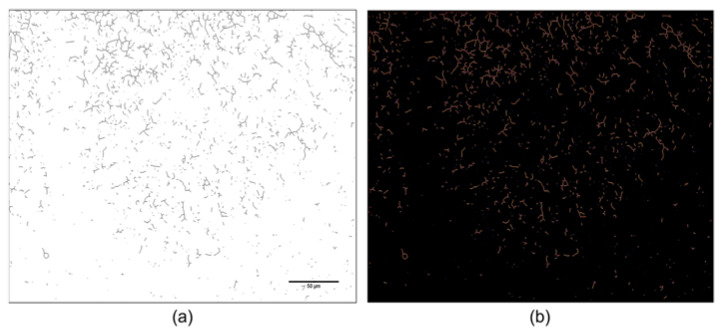
Representative images of the same photomicrographs through the processing steps. (**a**) Skeletonized mask; (**b**) Analyzed skeletonization. Bar = 50 µm.

Finally, Skeletonize [Process > Binary > Skeletonize] was applied (Figure 7a), followed by AnalyzeSkeleton [Plugin > Skeleton > AnalyzeSkeleton] (Figure 7b) [13].

## Figures and Tables

**Table 1:** Comparison between conventional analysis characteristics and semi-automatic method characteristics.

Conventional Analysis of Photomicrographs	Improved Semi-Automatic Method
Manual quantification	Macro-friendly, semi-automatic processing
User subjectivity	Plugin definition precision removes user subjectivity
Dependent on image quality and magnification	Applicable to low-magnification images
Complex photomicrographs	Independent of photomicrographs complexity
Inappropriate for large samplings	Appropriate for any sampling size
Time-consuming (manual quantification of photomicrographs from one treatment group may take weeks)	Fast (photomicrograph quantification from one treatment group, and data trimming done under one week)
Expensive hardware and /or software	Use of common hardware/software from a biology laboratory

## Data Availability

The data presented in this study are available on request from the corresponding authors. The data are not publicly available, due to privacy restrictions.

## References

[1] Barres B.A. (2008). The mystery and magic of glia: A perspective on their roles in health and disease. Neuron.

[2] Pekny M., Wilhelmsson U., Pekna M. (2014). The dual role of astrocyte activation and reactive gliosis. Neurosci. Lett..

[3] Ullian E.M., Christopherson K.S., Barres B.A. (2004). Role for glia in synaptogenesis. Glia.

[4] Winn H.R. (2022). Youmans and Winn Neurological Surgery.

[5] Crowe A.R., Yue W. (2019). Semi-quantitative determination of protein expression using immunohistochemistry staining and analysis: An integrated protocol. Bio-Protocol.

[6] Dias-Carvalho A., Ferreira M., Reis-Mendes A., Ferreira R., Bastos M.L., Fernandes E., Sá S.I., Capela J.P., Carvalho F., Costa V.M. (2022). Chemobrain: Mitoxantrone-induced oxidative stress, apoptotic and autophagic neuronal death in adult CD-1 mice. Arch. Toxicol..

[7] Sholl D. (1953). Dendritic organization in the neurons of the visual and motor cortices of the cat. J. Anat..

[8] Young K., Morrison H. (2018). Quantifying microglia morphology from photomicrographs of immunohistochemistry prepared tissue using ImageJ. J. Vis. Exp..

[9] Siritantikorn S., Jintaworn S., Noisakran S., Suputtamongkol Y., Paris D.H., Blacksell S.D. (2012). Application of ImageJ program to the enumeration of Orientia tsutsugamushi organisms cultured in vitro. Trans. R. Soc. Trop. Med. Hyg..

[10] Pekny M., Pekna M. (2004). Astrocyte intermediate filaments in CNS pathologies and regeneration. J. Pathol. J. Pathol. Soc. Great Br. Irel..

[11] Eroglu C., Barres B.A. (2010). Regulation of synaptic connectivity by glia. Nature.

[12] Rouach N., Koulakoff A., Abudara V., Willecke K., Giaume C. (2008). Astroglial metabolic networks sustain hippocampal synaptic transmission. Science.

[13] Arganda-Carreras I., Fernández-González R., Muñoz-Barrutia A., Ortiz-De-Solorzano C. (2010). 3D reconstruction of histological sections: Application to mammary gland tissue. Microsc. Res. Tech..

